# Type 2 Diabetes Mellitus Prevalence and Associated Risk Factors in Postmenopausal Women

**DOI:** 10.7759/cureus.60247

**Published:** 2024-05-14

**Authors:** D Varalakshmi, K Rekha, Rafi Mohammed

**Affiliations:** 1 Physiotherapy, Saveetha College of Physiotherapy, Saveetha Institute of Medical and Technical Sciences, Chennai, IND; 2 Physiotherapy, Apollo College of Physiotherapy, Hyderabad, IND; 3 Physiotherapy, School of Allied and Health Care Sciences, Malla Reddy University, Hyderabad, IND

**Keywords:** blood pressure, triglycerides, total cholesterol, waist circumference, body mass index, risk factors, postmenopausal women, type 2 diabetes mellitus

## Abstract

Introduction

Type 2 diabetes mellitus (T2DM) is the most common problem in postmenopausal women. This study aimed to find out the prevalence of T2DM and its risk factors in postmenopausal women.

Methods

The study is a population-based cross-sectional study. Anthropometric measurements, blood pressure, and biochemical measurements of 2295 postmenopausal women up to the age of 55 years were taken following face-to-face interviews. Odds ratio was used to find out the role of risk factors associated with T2DM.

Results

Prevalence of T2DM was reported to be 15.51%. Significant contribution of waist circumference (WC) followed by body mass index (BMI), total cholesterol (TC), and triglycerides (p<0.05) was noted in increasing the risk of T2DM. No association was found between T2DM and hypertension (p>0.05).

Conclusion

A high prevalence of T2DM was reported in postmenopausal women. Higher levels of BMI, WC, TC, and triglycerides were found to be the major risk factors for T2DM.

## Introduction

The clinical and economic burden associated with diabetes mellitus and its management remains an enduring challenge to the healthcare community [1]. It is well known that menopause status affects estrogen level [2] which plays an important role in regulating glucose homeostasis [3,4] and fat metabolism [5,6]. In menopause, a decrease in estrogen level caused by the depletion of ovarian function results in an increase in abdominal fat [7]. These alterations in body composition may possibly affect insulin sensitivity and glucose metabolism in postmenopausal women [8].

Type 2 diabetes mellitus (T2DM) is the most common type of diabetes mellitus, also known as insulin-resistant diabetes mellitus, a heterogeneous group of disorders characterized by different levels of impaired insulin secretion, insulin resistance, and increased production of glucose. In T2DM, either the insulin production is insufficient or the insulin cannot be used effectively by the body. Lower levels of the estrogen and progesterone hormones and also human growth hormone are some of the main reasons for T2DM in women over the age of 40. The insufficient production of the hormones contributes to lower metabolism and obesity which in turn is the main cause of T2DM [9].

Previously, several studies analyzed the association between menopause and T2DM. However, the analysis is not simple and straightforward because of multiple interrelating factors that may curtail the original clinical manifestations [10]. This study was undertaken to find out the prevalence of T2DM in postmenopausal women, to identify the risk factors for T2DM, and to assess its impact on the menopause age and the symptoms of climacteric disorders.

## Materials and methods

A population-based cross-sectional study was conducted involving 2295 postmenopausal women. The participants were the patients from the outpatient department who consulted in a medical center and the companions of the patients. Postmenopausal women up to the age of 55 years were included. Women who underwent any gynecological surgeries were excluded from the study. The study was conducted between July 2022 and January 2023. Approval was obtained from the Institutional Ethical Committee of Apollo Institute of Medical Sciences and Research, Hyderabad (approval number: EC/AIMSR/1527/2022/05/17).

Data collection

Data collection was done through face-to-face interviews followed by physical measurements and the collection of blood samples from the participants. The face-to-face interview was conducted using a questionnaire to collect information on age, educational status, marital status, occupation, and history of blood pressure and diabetes (see Appendices). Relevant equipment and procedures were used for anthropometric measurements, blood pressure, and biochemical measurements.

Height was measured in centimeters and weight was recorded in kilograms. Body mass index (BMI) was determined by height and weight measurements. The Asia-Pacific classification of BMI was used to categorize the participants into normal weight (BMI=18.5-22.9), overweight (BMI=23-24.9), and obese (BMI ≥25) [11]. A constant tension tape was used to measure the waist circumference (WC) as per the STEPS Manual guideline (WHO STEPwise approach to non-communicable disease (NCD) risk factor surveillance) [12]. A WC of 80 cm was taken as a cutoff point [13].

Participants were considered diabetic if the fasting glucose level was ≥126 mg or the postprandial blood glucose level was ≥200 mg or if they were already on anti-diabetic medication at the time of this study [14]. Doctor's aneroid sphygmomanometer was used to measure blood pressure. During the study, the participants were considered hypertensive if the systolic blood pressure was >140 mm of Hg and/or the diastolic blood pressure was >90 mmHg or if they were previously diagnosed as having hypertension.

Statistical analysis

Descriptive statistics was used to analyze the data. The values were expressed in mean, frequencies, and percentages. Odds ratio was used to find out the role of risk factors associated with T2DM. A p-value of <0.05 was considered statistically significant. GraphPad Prism 10.2 was used to perform all analyses.

## Results

A total of 2295 subjects participated in the study. The study findings revealed that the mean age of the participants was 51.18 years. Table 1 shows that 2274 out of 2295 participants (99.08%) were married. Many participants, i.e., 1629 out of 2295 (70.98%), were literate (completed high school education). Of the 2295, housewives were 1156 (50.37%). The BMI of 925 (40.3%) participants was ≥25 kg/m^2^. WC was also found to be higher than the normal levels in 949 (41.35%) participants.

**Table 1:** General and disease-specific characteristics of the subjects BMI: body mass index; WC; waist circumference

Characteristics	Frequency	Percent
Age group		
≤50	1709	74.46
>50	586	25.53
Educational status		
Completed schooling	1629	71
Did not complete schooling	666	29
Occupation		
Employees	1139	49.62
Housewives	1156	50.37
Marital status		
Married	2274	99.08
Unmarried	21	0.91
BMI		
≥25 kg/m^2^	925	40.30
<25 kg/m^2^	1370	59.69
WC		
≥80	949	41.35
<80	1346	58.64

Prevalence of T2DM was reported to be 15.51% (356 out of 2295). Table 2 presents the odds ratios of different variables in relation to T2DM in postmenopausal women. The risk of T2DM increased with BMI ≥25 (OR 2.79; p<0.0001). Increased risk of T2DM was also noted with WC (OR 4.52; p<0.0001), total cholesterol (TC) (OR 1.83; p<0.0001), and triglycerides (OR 1.70; p<0.0001). No significant association of T2DM was seen with increase in systolic blood pressures (OR 1.21; p=0.09) and diastolic blood pressures (OR 1.23; p=0.07).

**Table 2:** Risk of T2DM with other characteristics T2DM: type 2 diabetes mellitus; BMI: body mass index; WC: waist circumference; TG: triglycerides; TC: total cholesterol; SBP: systolic blood pressure; DBP: diastolic blood pressure *p<0.05 is statistically significant

Variable	Odds ratio	95% of CI	P-value^*^
BMI	2.79	2.2122-3.5232	<0.0001
WC	4.52	3.5329-5.8066	<0.0001
TC	1.83	1.4616-2.3036	<0.0001
TG	1.70	1.3567-2.1367	<0.0001
SBP	1.21	0.9685-1.5248	0.09
DBP	1.23	0.9824-1.5462	0.07

## Discussion

Our findings suggest that postmenopausal status can be considered as a well-founded and most important cause of T2DM and its prevalence in postmenopausal women has reached a distressing level. Menopause is linked with aging itself, and that is the reason why we limited our analyses to women aged up to 55 years. Many epidemiologic studies stated that impaired fasting glucose [15], dyslipidemia [16], and obesity [17] increase following menopause and all these metabolic disorders increase the risk of T2DM [18]. We found in our study that the risk of T2DM was strongly linked with postmenopausal status and the risk of T2DM with postmenopausal status was higher with BMI and WC. A decrease in estrogen level is considered to be the main cause of weight gain in women after menopause. A decrease in estrogen levels with menopause is also associated with an increase in central fat [19], and it has emerged as a risk factor for diabetes, hypertension, hypertriglyceridemia, and cardiovascular diseases [20]. Our results are consistent with the findings of a multinational study that found BMI and WC above normal levels were the risk factors for T2DM [21]. Our results also agree with the previous studies that assessed some of these parameters which are risk factors for diabetes. For instance, a Nigerian study [20] compared two groups of women with different levels of BMI and found that the risk for diabetes was higher in women with higher levels of BMI. In this way, menopause is followed by redistribution of adipose tissue to visceral depots and is associated with insulin resistance. It is widely accepted that being overweight or obese is predominantly related to diabetes. However, there is an increased risk of diabetes in Asians with a relatively lower BMI compared to the Western population [22]. The changes in body composition which take place after menopause are linked to impaired insulin sensitivity. It has also been demonstrated that abdominal obesity and impairments in both glucose tolerance [23] and insulin sensitivity [24] are associated independently among postmenopausal women. However, this relationship between abdominal obesity and insulin resistance has been well established even in the general population in several studies [25].

Other important risk factors of T2DM in postmenopausal women in our study were TC and triglycerides. These results are in line with previous studies. For example, the prevalence of T2DM was associated with dyslipidemia in a study conducted by Li et al. [26]. Another study indicated that the risk of diabetes increases with increased triglyceride levels [27]. It has been suggested in several epidemiologic studies that the incidence of dyslipidemia [16], obesity [15], and abnormal fasting blood glucose [17] increases in postmenopausal women and all these metabolic abnormalities are risk factors for T2DM [18]. Interestingly, no association was found between diabetes mellitus and hypertension in our study. Both systolic and diastolic blood pressures were not considerable risk factors for diabetes mellitus. These findings are quite different from what has been found in previous studies which demonstrated that the risk of diabetes mellitus increases with hypertension [19,26,27]. In a Women's Health study, it was suggested that hypertension is a strong predictor for the development of T2DM [28]. Hypertension was the main risk factor for diabetes mellitus in a study of a large number of women with climacteric symptoms [29]. It is well established that hypertension is very common in women with diabetes, since there is an extensive overlap between hypertension and diabetes in their etiology and mechanism of disease. This is because obesity leads to inflammation followed by insulin resistance and oxidative stress [30].

There has been a sharp increase in the prevalence of T2DM in postmenopausal women, and organizations have to gear up for urgent population-based interventions to prevent or delay its onset.

Study limitations

Our study limitation is that only a few variables were tested due to cost constraints.

## Conclusions

The findings of our population-based cross-sectional study demonstrate that the prevalence of T2DM is high among postmenopausal women. WC, BMI, TC, and triglycerides were found to be the major risk factors for T2DM in postmenopausal women.

## Appendices

Questionnaire

Name:

Age:

Height:

Weight:

1.     What is your highest qualification?

2.     What is your marital status?

3.     What is your current employment status? 

4.     What is your annual income?

5.     Have you ever been diagnosed with diabetes?

6.     Have you ever been diagnosed with hypertension?
